# Low-Frequency Oscillations in Cardiac Sympathetic Neuronal Activity

**DOI:** 10.3389/fphys.2020.00236

**Published:** 2020-03-18

**Authors:** Richard Ang, Nephtali Marina

**Affiliations:** ^1^Centre for Cardiovascular and Metabolic Neuroscience, Neuroscience, Physiology and Pharmacology, University College London, London, United Kingdom; ^2^Division of Medicine, University College London, London, United Kingdom

**Keywords:** sympathetic, arrhythmia, oscillations, cardiac repolarization, cardiac innervation

## Abstract

Sudden cardiac death caused by ventricular arrhythmias is among the leading causes of mortality, with approximately half of all deaths attributed to heart disease worldwide. Periodic repolarization dynamics (PRD) is a novel marker of repolarization instability and strong predictor of death in patients post-myocardial infarction that is believed to occur in association with low-frequency oscillations in sympathetic nerve activity. However, this hypothesis is based on associations of PRD with indices of sympathetic activity that are not directly linked to cardiac function, such as muscle vasoconstrictor activity and the variability of cardiovascular autospectra. In this review article, we critically evaluate existing scientific evidence obtained primarily in experimental animal models, with the aim of identifying the neuronal networks responsible for the generation of low-frequency sympathetic rhythms along the neurocardiac axis. We discuss the functional significance of rhythmic sympathetic activity on neurotransmission efficacy and explore its role in the pathogenesis of ventricular repolarization instability. Most importantly, we discuss important gaps in our knowledge that require further investigation in order to confirm the hypothesis that low frequency cardiac sympathetic oscillations play a causative role in the generation of PRD.

## Introduction

Sudden cardiac death caused by ventricular arrhythmias is a leading cause of mortality globally, resulting in approximately 50% of all cardiovascular-related deaths each year (Wong et al., 2019). Excessive sympathetic activity is a crucial factor known to promote myocardial repolarization abnormalities that increase the vulnerability of developing ventricular fibrillation and fatal cardiac arrhythmias (Maling and Moran, 1957; Cao et al., 2000). Recent studies have shown that ventricular repolarization instability after an acute myocardial infarction (MI) exhibits a pronounced rhythmic pattern that is believed to mimic the characteristic low-frequency (LF) oscillations in sympathetic efferent activity (Rizas et al., 2014, 2017; Pueyo et al., 2016). This electrophysiological phenomenon has been termed periodic repolarization dynamics (PRD) and it can be measured non-invasively from the vector angle of the electrocardiogram (ECG) T wave (Rizas et al., 2014).

This article aims to review the available evidence in support of the hypothesis that rhythmic sympathetic nerve traffic to the myocardium underlies the origin of PRD. We first focus on how excessive adrenergic signaling affects myocardial repolarization. We then present a comprehensive review of published data obtained in rodent brain tissue *in vitro* and in whole animal preparations *in vivo* that prove that sympathetic neuronal networks most likely involved in the control of cardiac function can exhibit LF oscillatory activity under some experimental conditions. We then discuss the mechanisms involved in the generation of rhythmic sympathetic discharges and explore the physiological role of patterned activity in the sympathetic system and its impact on neurotransmission efficacy. In doing so, we identify important gaps in our knowledge that need to be addressed in future studies.

### Cardiac Ventricular Repolarization and the Surface ECG T Wave

Cardiomyocyte repolarization represents a complex sequence of electrical events that occur during phases 1 to 3 of the action potential in which the net outward current exceeds the net inward current, causing the return of the membrane potential to its baseline resting state prior to the next depolarization. Cardiac ventricular repolarization is a significant determinant of the QT interval, represented on the surface ECG by the interval between the start of the QRS complex and the end of the T wave (Yan et al., 2003).

There is general agreement that the T wave is the result of voltage gradients that exist within the ventricular myocardium during cardiac repolarization although the precise mechanism appears to differ depending on the species studied and the experimental preparation used. Using arterially perfused canine right ventricular wedge preparations, Antzelevitch found three layers of electrically and functionally distinct cell types of the ventricular myocardium: the epicardial cells, the M cells and the endocardial cells (Antzelevitch, 2006). These studies demonstrated that the T wave arises due to transmural voltage gradients across the ventricular myocardium which develop as a result of the difference in the time course of repolarization of the three layers, with the M cells having the longest action potential duration (APD) followed by the endocardial layer and the epicardial layer. However, mapping studies using arterially perfused left ventricular wedge preparations and intact hearts suggest that transmural repolarization differences do not fully explain T wave genesis (Opthof et al., 2007; Boukens et al., 2015). By comparing electrical and optical mapping of both intact and left ventricular wedge preparation of canine hearts, Boukens et al. (2017) demonstrated that electrical gradients from wedge preparations differed from those of intact hearts, implying that findings from wedge preparations may not extrapolate to the whole heart.

In addition to the transmural electrical gradient, there is also evidence of electrical heterogeneity between the apex to base (Autenrieth et al., 1975; Watanabe et al., 1985; Franz et al., 1987) and left to right ventricles of the heart (Durrer et al., 1970; Srinivasan et al., 2016). Indeed, whole heart studies have shown that the T wave is an index of dispersion of repolarization across the whole heart and not due to transmural electrical gradients (Meijborg et al., 2014; Opthof et al., 2017; Srinivasan et al., 2019).

### Effects of Sympathoexcitation on Cardiac Repolarization and Ventricular Arrhythmia

Sympathoexcitation leads to norepinephrine release which activates β-adrenoceptors (β-AR) to modulate myocardial repolarization and contractility. β-AR stimulation increases L-type Ca^2+^ current which leads to an increase in APD but this is counterbalanced by the concomitant increase in outwards K^+^ currents via the rapidly (*I*_Kr_) and slowly (*I*_Ks_) activating delayed rectifier potassium channels (Hartzell, 1988). Sympathoexcitation can lead to both APD shortening or prolongation depending on the net effect of the inwards and outwards currents (Priori and Corr, 1990). This effect is species-dependent and in humans it has been shown to lead to APD prolongation (Jakob et al., 1988; Veldkamp et al., 2001).

The arrhythmogenic effects of excessive noradrenergic tone are exerted at different levels. At the cellular level, β-AR activation leads to cyclic AMP (cAMP) dependent phosphorylation of proteins involved in excitation–contraction coupling which includes L-type Ca^2+^ channels, ryanodine receptors (RyR) and phospholamban, with concomitant increase in sarcoplasmic reticulum (SR) Ca^2+^-ATPase (SERCA) activity (Hartzell, 1988). This results in an increase in cytosolic and SR Ca^2+^ levels which can result in a triggered action potential via Na^+^/Ca^2+^ exchanger (NCX) and membrane depolarizations during phase 4 of the action potential. This process is also known as delayed after depolarizations (DADs) (Pogwizd and Bers, 2004) and pathological processes such as MI and subsequent heart failure are believed to increase the likelihood of DADs by inducing an increase in the expression of NCX (Pogwizd et al., 1999) and by promoting SR Ca^2+^ leak via RyR (Shannon et al., 2003) and a decreased *I*_Kr_ current (Pogwizd et al., 2001). At the tissue level, a further requirement for arrhythmogenesis is electrical coupling between the focus of origin and the surrounding tissue (Kumar et al., 1996). Electrical coupling through gap junctions silences “unstable” tissue by the surrounding “stable” cells (also described as “source-sink” effect) (Xie et al., 2010). DADs occurring simultaneously in several thousand cells is hence required to generate enough depolarizing current to produce a propagating action potential, which is manifested clinically as premature ventricular complexes (PVCs) (Myles et al., 2012). Adverse remodeling secondary to disease processes can lead to decreased gap junction coupling which results in a lower number of cells with DADs required to generate an abnormal impulse (Poelzing and Rosenbaum, 2004). On a macro level, overt tissue fibrosis can also result in areas of electrically unexcitable tissue which creates the condition for re-entrant arrhythmias to occur (Vaquero et al., 2008). Finally, at the whole heart level, the heterogeneous distribution of sympathetic nerves across the heart may also play an important role in the generation of ventricular arrhythmias. The density of sympathetic nerve terminals appears to be more abundant at the ventricular base compared to the apex and these regional differences may have a profound effect on the APD gradient from endocardial to epicardial layers (Nabauer et al., 1996; Brunet et al., 2004; Ieda et al., 2007; Lorentz et al., 2010). As a result, even under non-pathological conditions, sympathetic activation would lead to non-uniform changes in APD across the ventricles, increasing the dispersion of repolarization and the potential for re-entrant arrhythmias. In pathological conditions (i.e., diabetes, obesity, MI, and heart failure), where maladaptive cardiac sympathetic innervation remodeling occurs, heterogeneity of APD and repolarization may become even more pronounced (Gardner et al., 2016).

In summary, sympathoexcitation leads to both an increase in triggered activity, and dispersion of repolarization. This leads to abnormalities in activation and propagation of electrical activity in the ventricular myocardium that have been shown in experimental and clinical studies to be pro-arrhythmic (Maling and Moran, 1957; Cao et al., 2000).

### Low Frequency Oscillation T Wave Dynamics as a Marker of Sympathoexcitation and Susceptibility to Ventricular Arrhythmia

An area of intense clinical research has been the search for a reliable biomarker of increased susceptibility to potentially fatal ventricular arrhythmia which would help direct clinical intervention such as prophylactic implantation of an implantable cardioverter defibrillator device (ICD). Various non-invasive methods have been studied, including assessments of increased/altered sympathetic tone [heart rate variability (Schmidt et al., 1999) and baroreflex sensitivity (Billman et al., 1982)] and measurements of abnormalities in cardiac repolarization [QT interval (Zhang et al., 2011), QT dispersion (Day et al., 1990), Tpeak to Tend (Panikkath et al., 2011) and microvolt T wave alternans (Verrier et al., 2011)]. However, these methods are not accurate as they only provide an indirect probe of the sympathetic effect on cardiac repolarization. For example, the effect of autonomic tone on sinus node activity is not excluded when studying QT interval as the heart rate is not kept constant and changes in heart rate in itself may affect the QT interval. Furthermore, there may also be concomitant influences of the sympathovagal tone on the vasculatures and on the renin-angiotensin-aldosterone system which may confound the interpretation of the results.

Periodic repolarization dynamics has been proposed as a promising risk marker for susceptibility to ventricular arrhythmia. PRD is assessed using a high resolution ECG recorded in ‘Frank lead configuration’ with three orthogonal axes *X*-, *Y*-, and *Z*-. Low frequency (≤0.1 Hz) periodic changes of the T wave vector provide an index to measure both sympathetic activity and its effects on ventricular repolarization. In a cohort of 908 patients, increased PRD predicted total and cardiovascular mortality in survivors of MI and was independent of underlying heart rate and respiratory activity. Furthermore, in multivariate analysis PRD provided incremental prognostic value in addition to established risk markers such as LV ejection fraction and measure of T wave alternans (Rizas et al., 2014). A recent 5-year prospective multicenter study (EUropean Comparative Effectiveness Research to Assess the Use of Primary ProphylacTic Implantable Cardioverter Defibrillators, EU-CERT-ICD) showed a strong correlation between the magnitude of the oscillations of ventricular repolarization with both arrhythmia and sudden death in patients with ischemic and non-ischemic cardiomyopathy. Thus, PRD has great potential as a clinical tool for risk stratification of patients who would benefit from implantation of implantable cardioverter defibrillators (Bauer et al., 2019).

The link between PRD and the level of sympathetic activity was demonstrated in clinical studies showing that manipulations that trigger sympathoexcitatory responses (i.e., exercise or the tilt test) enhance the magnitude of PRD whilst pharmacological blockade of β-adrenergic antagonists have the opposite effect (Rizas et al., 2014). The periodicity of the oscillations in ventricular repolarization appears to be in the same frequency range of the LF oscillatory patterns detected in muscle sympathetic nerve activity recordings (MSNA) (Furlan et al., 2000) and in the spontaneous beat-to-beat oscillations in the R–R interval (RRi) (Pagani et al., 1986; Malliani et al., 1991). Other studies have also demonstrated similar rhythmic patterns of APD in patients with heart failure which are coherent with the 0.1 Hz oscillatory frequency of arterial blood pressure Mayer waves (Hanson et al., 2014). Similarly, a recent study has shown that LF oscillations in APD were reduced following β-ADR blockade and were correlated with changes in RRi (Duijvenboden et al., 2019). Together, this evidence has led to the suggestion that LF oscillatory patterns in APD and PRD represent the effect of sympathetic nerve activity on the myocardium (Rizas et al., 2014, 2016). However, this hypothesis is based on associations of PRD with indirect measurements of sympathetic activity which are not anatomically or functionally involved in the regulation of ventricular excitability and repolarization: first, RRi is believed to represent sympathetic influences on the sino-atrial node (Malliani et al., 1991) which explains why PRD is not affected when heart rate variability is eliminated in subjects with fixed atrial pacing (Rizas et al., 2014). Second, arterial blood pressure Mayer waves are believed to result from rhythmic oscillations of muscle vasoconstrictor activity (Julien, 2006). Third, MSNA is a direct measurement of sympathetic vasomotor tone that is usually recorded at the peroneal nerve (Macefield, 2013). Thus, in the following sections we have sought to identify experimental evidence of the existence of oscillatory activity in neuronal networks and peripheral nerves along the neurocardiac axis which might have a more direct role in the regulation of ventricular myocardial excitability.

### Rhythmic Sympathetic Activity

One of the most remarkable characteristics of sympathetic neuronal discharges is their rhythmic nature. Autonomic neuroscientists have applied power spectral analysis methods based on fast Fourier transform (FFT) algorithms to detect rhythmic patterns in sympathetic neurons and peripheral nerves both in experimental laboratory animals and in human subjects (Montano et al., 2009).

Rhythmic sympathetic oscillations occur over a wide spectrum of distinct frequencies, ranging from 0.1 to 10 Hz, depending on the sympathetic outflow being measured (Malpas, 1998). In humans, a LF rhythm (≤0.1 Hz) is often found in direct recordings of MSNA (Furlan et al., 2000) and in the variability of heart rate (HR) and systolic arterial blood pressure (SAP) (Pagani et al., 1986; Malliani et al., 1991). However, these sympathetic outflows are unlikely to have a direct role in the control of cardiac excitability since they are not anatomically linked with the innervation of ventricular cardiomyocytes and their control mechanisms may differ from the systems that control cardiac sympathetic outflow. Since direct measurements of cardiac sympathetic outflows cannot be investigated in human subjects, we will primarily discuss evidence obtained in experimental laboratory animals using invasive techniques for the direct assessment of cardiac sympathetic neuronal activity (CSNA).

Low-frequency oscillations in sympathetic outflows appear to be less ubiquitous than cardiac-related (2–6 Hz) and respiratory-related (1–3 Hz) rhythms. Nevertheless, numerous studies have found that LF rhythms are a robust feature of sympathetic neuronal networks involved in the control of cardiac function. Although none of the studies discussed in the following sections have investigated rhythmic sympathetic oscillations in the context of ventricular repolarization instability, the mechanisms described herein are likely to contribute, at least in part, to the origin, regulation and synchronization of PRD.

### LF Oscillations in Brainstem Neuronal Circuits

Sympathetic activity originates in a lower brainstem region known as the rostral ventrolateral medulla (RVLM). The RVLM contains a group of C1 catecholaminergic neurons and a group of non-catecholaminergic neurons believed to produce glutamate (Brown and Guyenet, 1985; Schreihofer and Guyenet, 1997; Guyenet, 2006). RVLM neurons send monosynaptic excitatory inputs to sympathetic preganglionic neurons (spns) within the thoraco-lumbar spinal cord (Amendt et al., 1979; Ross et al., 1981) that are crucial for the maintenance of resting vascular tone and heart rate (Marina et al., 2011).

Low-frequency oscillatory patterns have been identified in single RVLM neurons in experiments conducted in unanesthetized, decerebrated, vagotomised and artificially ventilated cats with denervated baroreceptors (Montano et al., 1995, 1996) and in rats anesthetized with urethane with either intact or denervated baroreceptors (Tseng et al., 2009). RVLM neuronal LF oscillations were shown to be correlated with the LF component of the systolic arterial pressure variability (Montano et al., 1995) and were found to be involved in the generation of coherent LF oscillations in renal sympathetic nerve outflows (Tseng et al., 2009). Although the identity of the target organ innervated by these neurons was not identified in these studies, these data strongly suggest that LF sympathetic oscillations have a central origin as they were detected in the absence of cardio-respiratory and baroreceptor inputs. Experiments conducted in adult decerebrated cats have also identified the presence of 0.1 Hz oscillatory activity in pontine neurons involved in respiratory pattern generation and in medullary raphé neurons that modulate both, sympathetic nerve activity and the activity of brainstem respiratory networks (Morris et al., 2010). LF oscillations in pontine and raphé neurons were coordinated with arterial blood pressure Mayer waves and became synchronized with the central respiratory rhythm after elimination of pulmonary stretch receptor inputs (Morris et al., 2010). Together, these results suggest that LF oscillatory activity originates in a dispersed supraspinal neuronal network that participates in the integration of vasomotor, cardiac-related and respiratory rhythms.

Little is known about the cellular mechanisms that contribute to the origin of LF oscillations in supraspinal neuronal networks. At the single cell level, *in vitro* studies have demonstrated that RVLM neurons have the capability of displaying intrinsic pacemaker activity in conditions of reduced synaptic activity, which suggests that synaptic inputs are only involved in the modulation of rhythmic patterns (Sun et al., 1988a, b). Intracellular recordings conducted in retrogradely identified cells in isolated neonatal spinal cord preparations confirmed that a population of non-adrenergic reticulospinal neurons in the RVLM possess pacemaker-like properties such as an after-hyperpolarization at the end of the spike followed by a slow depolarization with no evidence of excitatory postsynaptic potentials (EPSPs) between action potentials (Sun et al., 1988b). However, single cell recordings in intact preparations (anesthetized rats) failed to support this “pacemaker hypothesis,” arguing that pacemaker-like activity recorded in RVLM neurons results from the anatomical or functional elimination of synaptic inputs (Lipski et al., 1996). Thus, a “network” hypothesis has been suggested which proposes that 2- to 6-Hz and 10-Hz oscillatory activities in medullary neurons originate from the influence of synaptic influences from neighboring brainstem oscillators located in the lateral tegmental field (LTF) which can be entrained by baroreceptor inputs (Barman and Gebber, 1987, 1993). Although these results illustrate some general mechanisms underlying the generation of rhythmic activity in bulbospinal neurons, they highlight the lack of evidence that might explain how cardiac presympathetic neurons generate LF oscillatory patterns, in particular in conditions of enhanced sympathetic drive.

### LF Oscillations in Sympathetic Preganglionic Neurons of the Spinal Cord

Neurons located across four distinct regions of the thoracolumbar spinal cord integrate descending excitatory inputs from the RVLM and other sympathoexcitatory areas in the hypothalamus. These centers include the intermediolateral cell column (IML), nucleus intermediolateralis thoracolumbalis pars funicularis, intercalated nucleus (IN), and central autonomic area (CA). The axon from the spns exit the spinal cord through the ventral root to make synaptic contact with cardiac sympathetic ganglia via white rami communicans. Anatomical tracing studies in guinea pigs (Dalsgaard and Elfvin, 1981) and cats (Chung et al., 1975, 1979) have shown that spns that control cardiac function are mainly distributed along lower cervical and upper thoracic spinal segments (C8-T11).

Most of the evidence showing LF oscillatory activity in preganglionic neuronal networks comes primarily from *in vivo* studies conducted in whole animal preparations. Neuronal recordings from thoracic preganglionic axons in anesthetized cats with high spinal transection at the C1 level showed neuronal discharge variability in the range of 0.1 Hz that were temporally synchronized with the oscillations in systemic arterial pressure (Fernandez de Molina and Perl, 1965). Similar recordings conducted in decerebrated, unanesthetized cats confirmed the presence of rhythmic neuronal discharges in the LF range at the level of the third thoracic (T3) white ramus communicans that correlated with the LF component of the R–R interval (Lombardi et al., 1990; Montano et al., 1992). The power of LF oscillations was increased in response to a fall in systemic blood pressure and conversely, was decreased in response to elevations in arterial blood pressure (Montano et al., 1992). In a subsequent study Montano et al. (2000) found that LF preganglionic neuronal oscillations were preserved following acute spinal transection at the C1 level and the rhythmic discharges remained synchronized with the variability of the R–R interval and systolic blood pressure (Montano et al., 2000). In these conditions, the power of LF sympathetic discharges in preganglionic fibers innervating the stellate ganglion was found to increase in response to increases in arterial blood pressure and this effect was abolished when cardiovascular afferent inputs to the spinal cord were physically interrupted by a dorsal rhizotomy (Montano et al., 2000). Together, these data suggest that LF oscillations are generated locally within preganglionic sympathetic neuronal circuits and that positive-feedback spinal reflexes play an important role in the potentiation of LF oscillatory activity in cardiac-related spns.

The rhythmic properties of spns neuronal discharge have been studied primarily *in vitro* using acute spinal cord slices and isolated spinal cord preparations from neonatal rats. These studies have shown that the mechanisms underlying the generation of this rhythmic pattern result from a complex interaction between intrinsic membrane properties in individual neurons, synaptic inputs and network interactions within the spinal cord. At the single cell level, patch-clamp recordings in neonatal rat spinal cord slices revealed the presence of spontaneous membrane potential oscillations in spns independent of excitatory or inhibitory synaptic inputs, which suggests that oscillatory activity arises from intrinsic membrane properties in spns (Spanswick and Logan, 1990; Shen et al., 1994). At the network level, synchronized sympathetic activity is believed to emerge as a consequence of transmission of spontaneous membrane potential oscillations between gap-junction-coupled spns (Logan et al., 1996; Nolan et al., 1999). In support of this network hypothesis, our immunohistochemical studies have revealed that thoracic spns express connexin-36 proteins along somato-dendritic sites of close apposition (Marina et al., 2008) and pharmacological studies in spinal cord slices have shown that blockade of gap junctions attenuates and in some cases abolishes rhythmic activity in spns (Pierce et al., 2010).

Synaptic mechanisms have also been implicated in the generation of rhythmic activity in spns. Several studies have shown that rhythmic oscillations can be induced pharmacologically by activation of 5-HT receptors in spinal cord preparations *in vitro* (Pickering et al., 1994; Lewis and Coote, 1996; Pierce et al., 2010) and in *in situ* “isolated spinal cord preparations” in anesthetized rats (Marina et al., 2006). This suggests that oscillatory activity in preganglionic neuronal networks is generated in response to direct descending serotonergic excitatory inputs from the medulla oblongata (Smith et al., 1998).

Although rhythmic sympathetic activity in the spinal cord has received significant research attention, very little information is available about the putative mechanisms that give rise to the generation of LF oscillations in spns (Su, 2001; Sourioux et al., 2018). Electrophysiological recordings of preganglionic fibers innervating the celiac ganglion revealed the presence of spontaneous bursting activity in the range of <0.1 Hz that was abolished in the presence of a high Mg^+^ solution and was attenuated by application of non-NMDA receptor blockers (Su, 2001). A recent study showed that activation of muscarinic cholinergic receptors (mAchRs) trigger LF oscillatory activity in spns in coordination with somatomotor neuronal activity (Sourioux et al., 2018). These studies have thus identified cholinergic and glutamatergic neurotransmission mechanisms that play an important role in the generation of LF oscillations in spns that innervate visceral organs. Future studies should determine whether similar mechanisms operate in upper thoracic spinal segments which may facilitate the generation of LF oscillatory activity in sympathetic preganglionic networks that control the electrical activity of the heart.

### LF Oscillations in Cardiac Sympathetic Postganglionic Fibers

Extra-cardiac sympathetic neurons are located across numerous thoracic ganglia in particular in the stellate, middle cervical, superior cervical and mediastinal ganglia (Janes et al., 1986; Ardell and Armour, 2016). The electrophysiological properties of neurons located within the stellate ganglion and postganglionic axons traveling along the cardiac sympathetic nerve (CSN) have been studied extensively in several animal models *in vivo*, including ambulatory cats (Tsuchimochi et al., 2002), dogs (Han et al., 2012; Chan et al., 2015) sheep (Jardine et al., 2002, 2005, 2007; Charles et al., 2018) and anesthetized cats (Nishikawa et al., 1994). Experiments conducted in conscious and anesthetized cats with either intact or denervated baroreceptors have been the model of choice to study periodic oscillations in CSNA. However, the frequency of rhythmic neuronal oscillations reported so far appears to be dominated by cardiac-related rhythms in the 2-to 6-Hz range (Ninomiya et al., 1989, 1990, 1993; Kocsis et al., 1990; Hedman et al., 1994; Kocsis, 1994; Hedman and Ninomiya, 1995; Kocsis and Gyimesi-Pelczer, 1998; Larsen et al., 2000) or by respiratory-related rhythms in synchrony with the discharge frequency of the phrenic nerve (Kollai and Koizumi, 1980). The presence of LF oscillations in cardiac postganglionic fibers has not been documented yet, which appears to be counterintuitive, since the final synaptic relay in the neurocardiac axis would be expected to follow the same oscillatory pattern generated by either bulbar presympathetic or spinal preganglionic neuronal networks. However, this apparent lack of scientific evidence does not preclude the existence of LF oscillations in cardiac postganglionic fibers, as this might be the consequence of the experimental conditions used to obtain the data. As mentioned previously, LF oscillations in preganglionic axons have only been detected in decerebrated cats in the absence of general anesthesia (Montano et al., 1992, 2000). Previous studies have shown that anesthetic drugs produce a profound suppression of cardiac sympathetic nerve activity in cats (Matsukawa et al., 1993). This suggests that LF oscillations might have been suppressed and therefore were probably not detected in the studies where animals were anesthetized with chloralose (Kollai and Koizumi, 1980; Kocsis et al., 1990; Kocsis, 1994), sodium pentobarbital (Hedman et al., 1994) or urethane (Kocsis, 1994; Larsen et al., 2000). Another factor that might have interfered with the detection of LF oscillations in cardiac postganglionic fibers is the signal processing methods used for the discrimination of rhythmic components. In the decerebrated cat, cardiac preganglionic fiber power spectra are primarily dominated by a 3.3 Hz component which relates to the rhythm synchronous with the cardiac cycle. In contrast, the power spectral density of LF components in the 0–0.5 Hz range in these preparations is only minor (Lombardi et al., 1990). In order to better identify synchronized preganglionic activity in the LF range, cardiac synchronous rhythmicity needs to be eliminated by filtering the signal with low pass filters with a cut-off frequency of 1 Hz. This allows sampling of the neural signal once per-heart beat and application of autoregressive modeling analysis of sympathetic nerve discharges and R–R interval duration (Lombardi et al., 1990; Montano et al., 1992, 2000). In contrast, the studies assessing cardiac postganglionic nerve discharges cited above did not take measurements to eliminate cardiac-related synchronous activity which may have prevented the detection of LF oscillations (Kocsis et al., 1990; Kocsis, 1994; Kocsis and Gyimesi-Pelczer, 1998; Larsen et al., 2000).

### LF Oscillations in Intrinsic Ganglionated Cardiac Plexus

The intrinsic innervation of the heart includes a heterogeneous collection of sympathetic (Moravec and Moravec, 1989; Moravec et al., 1990) and parasympathetic (Yuan et al., 1993) cardiac ganglia collectively termed ganglionated plexus (GP). According to the classification proposed by Beaumont et al. (2013), intracardiac local circuit neurons (LCNs) fall into three main categories: (a) secondary afferent LCNs that detect mechanical and chemical stress signals from different regions of the heart, (b) secondary efferent LCNs that respond to sympathetic and/or parasympathetic neuronal inputs from higher brain centers, medullary networks, spinal preganglionic neurons and thoracic extracardiac ganglia, and (c) convergent LCNs that integrate afferent sensory information with efferent autonomic inputs. LCNs are believed to generate coordinated responses that control chronotropic, dromotropic, inotropic, and lusotropic properties of the heart (Armour, 1997, 2004; Armour et al., 1998).

*In vivo* studies conducted in anesthetized dogs showed that GP neurons residing in the ventral ventricular GP display spontaneous activity in synchrony to the cardiac cycle and the respiratory rhythm and are exquisitely sensitive to stimulation of β-adrenergic receptors and mechanical stimulation (Ardell et al., 1991). A subsequent study also found that epicardial application of voltage-gated sodium channel agonist veratridine induced robust bursting discharges in the range of 0.1 Hz in both, intrinsic ventricular GP neurons and extrinsic cardiac neurons. However, neuronal discharges were frequently found to be out of synchrony (Armour et al., 1998). In some experiments, transection of sympathetic and vagal neuronal connections also resulted in the generation of bursting activity in intrinsic and middle cervical ganglia neurons (Armour et al., 1998). However, manipulations to produce mechanical activation of carotid sinus baroreceptors failed to produce a significant activation of intrinsic cardiac neurons. These data suggest that LF bursting patterns are primarily displayed by ventricular secondary afferent intracardiac LCNs in response to the detection of chemical clues that are normally released in situations of myocardial damage. Future studies should aim to determine whether LF rhythmic activity generated by intracardiac LCNs is mechanistically linked to the facilitation of LF repolarization instability as observed in PRD.

### Cardiac Sympathetic Oscillations Post-MI

Clinical studies have shown that LF oscillations in the variability of muscle sympathetic nerve activity (MSNA) are significantly increased in patients after MI (Martinez et al., 2011). However, sympathetic vasoconstrictor fibers innervate arterioles that determine peripheral resistance which have no anatomical connection with regional cardiac targets. MSNA might be different to CSNA in terms of the regulation of central mechanisms underlying the generation of oscillatory patterns. Thus, increased MSNA is unlikely to be directly linked to the periodic fluctuations in cardiac repolarization observed in patients post-MI (Rizas et al., 2014).

To our best knowledge, there are no studies in experimental animals models of MI that have investigated changes in the power of the LF component in the variability of neuronal discharges of bulbospinal RVLM neurons, spns in the IML and postganglionic sympathetic neurons of the CSN. Electrophysiological studies conducted to investigate the neuronal response properties to MI in anesthetized cats have shown that interruption of the left coronary artery blood flow produced a substantial increase in the discharge of afferent sympathetic fibers that supply the ventricular myocardium and this effect was mimicked by intracoronary administration of bradykinin (Lombardi et al., 1981). Functional changes in sympathetic innervation are thus likely to contribute to post-MI hypersensitivity and may eventually lead to the loss of sympathetic fibers within the infarcted myocardium.

Mi triggers a cardio-cardiac reflex which results in increased activity of preganglionic fibers of the third thoracic white ramus communicans and these responses were found to be preserved in animals with spinal cord transection (Malliani et al., 1969). Experiments in conscious cats (Ninomiya et al., 1986) sheep (Jardine et al., 2005) and dogs (Han et al., 2012) have shown that neuronal discharges of the cardiac postganglionic neurons in the stellate ganglion and CSN increase significantly immediately after MI. Chronic stellate ganglion nerve activity (SGNA) recordings in ambulatory dogs revealed that increased neuronal excitability was observed in viable recordings for as long as 2 months and these changes were associated with increased nerve density at the stellate ganglion (Han et al., 2012).

A recent study using a porcine model of MI revealed that the spontaneous LF rhythmic firing rate of intracardiac GP neurons in the left ventricle are preserved at the same level following a MI, however, with a significant reduction in the detection of afferent inputs. These functional changes were associated with a significant increase in intracardiac neuron cell size and an upregulation in the expression of the sympathetic neuronal marker Tyrosine Hydroxylase (Rajendran et al., 2016). Future preclinical studies should investigate further whether periodic oscillations in ventricular repolarization in subjects post- MI are related to the function of intracardiac PG neurons.

### Functional Significance of Cardiac Sympathetic Oscillatory Activity

The physiological meaning of sympathetic oscillatory activity remains unclear. Many studies support the notion that rhythmic activity promotes the coordination of neuronal firing of individual sympathetic neurons which may lead to a highly coordinated and more efficient release of neurotransmitter at the nerve terminal (Nilsson et al., 1985; Ando et al., 1993; Janssen et al., 1997; Lisman, 1997; Dibona and Sawin, 1999). Also, rhythmicity is believed to allow the coordination of nerve discharges between different sympathetic outflows which may help to generate integrated responses that help maintain homeostasis (Barman and Kenney, 2007).

At present, the physiological mechanisms underlying the genesis of PRD are unknown. In this review article we worked under the unverified assumption that recurrent periods of ventricular repolarization instability follow the LF rhythm of cardiac sympathetic postganglionic activity. In support of this hypothesis, *in vitro* studies conducted in fully innervated isolated rabbit hearts have shown that electrical stimulation of CSN (with a stimulation frequency of 15 Hz for 50 s) changed the spatial dispersion of repolarization (DOR) from apex toward the base to base toward the apex within 15 s following the start of the stimulation (Mantravadi et al., 2007). When sympathetic nerve stimulation was interrupted, DOR returned slowly to baseline levels and to its original direction (i.e., apex to base) after approximately 2 min. Although the dynamics of the responses obtained under these experimental conditions (50 s stimulation) make it difficult to extrapolate these results with the periodicity of PRD reported to occur every 10 s (Rizas et al., 2014, 2016), these data provide important clues about the transfer function between neurotransmitter release from the cardiac sympathetic terminals and the concomitant changes in cardiomyocyte repolarization (Mantravadi et al., 2007). Further work needs to be done using similar experimental models but providing rhythmic bursts of sympathetic stimulation to conclusively determine whether LF oscillatory cardiac sympathetic activity translates into periodic oscillations of ventricular repolarization instability.

## Conclusion

Since Adrian et al. (1932) published their seminal study on rhythmic spontaneous bursting activity in sympathetic nerves of anesthetized animals, a considerable amount of literature has been generated describing the features, mechanisms and possible physiological implications of sympathetic rhythmic activity. However, the translational potential of this mechanism has remained obscure for almost a century.

The discovery of PRD as a novel marker of CSN traffic and strong predictor of death has recently reignited the interest in the phenomenon of sympathetic rhythmicity. The experimental evidence reviewed here identified sympathetic circuitries contained in the brainstem and in the spinal cord which may have direct connections with the ventricular myocardium and that are capable of generating LF oscillatory activity. However, the hypothesis that PRD is directly driven by sympathetic neuronal oscillations (Rizas et al., 2014, 2016) is still missing crucial pieces of evidence, specifically: (i) can postganglionic sympathetic fibers to the heart exhibit LF oscillations, in particular in conditions associated with increased cardiac sympathetic tone?, (ii) are LF sympathetic oscillations themselves pro-arrhythmogenic?, and (iii) is it the amplitude of PRD oscillations what determines its arrhythmogenic potential? The latter is particular relevant since PRD oscillations can be detected in healthy individuals and their amplitude can be increased in response to pharmacological and physiological sympathoexcitatory interventions (Rizas et al., 2014). However, physiological increases in cardiac sympathetic tone and therefore increases in the amplitude of PRD oscillations in healthy subjects do not appear to have arrhythmogenic effects. In contrast, in a cohort of post-MI patients who did not survive the 5-year follow up period, the amplitude of PRD oscillations appears to be much greater than in surviving patients (Rizas et al., 2014). This suggests that the myocardium in non-survivors is more vulnerable to the arrhythmogenic effects of sympathetic oscillatory activity. Alternatively, we speculate that in post-MI subjects with high mortality risk, increased amplitude of PRD oscillations might reflect a higher degree of synchronization among cardiac presympathetic and preganglionic neurons which would allow the recruitment of previously silent postganglionic fibers that innervate specific targets within the heart, such as the myocardium. This would result in the release of copious amounts of norepinephrine from the sympathetic terminals and perhaps the release of arrhythmogenic co-transmitters such as Neuropeptide Y (Kalla et al., 2019) which may ultimately precipitate profound periodic changes in ventricular repolarization.

## Author Contributions

NM designed the review and drafted the manuscript. RA contributed to the writing of the final version of the manuscript. Percentage contributions are NM 70% and RA 30%. All authors read and approved the final manuscript.

## Conflict of Interest

The authors declare that the research was conducted in the absence of any commercial or financial relationships that could be construed as a potential conflict of interest.
